# Foreign Body in the Bronchus Intermedius: Inadvertent Deployment of a Bravo Wireless pH Probe in the Airway

**DOI:** 10.1097/PG9.0000000000000299

**Published:** 2023-03-13

**Authors:** Steven M. Andreoli, Stefanie Schrum, Katherine McGoogan

**Affiliations:** From the * Division of Pediatric Otolaryngology, Nemours Children’s Health, Jacksonville, FL; † Department of Pediatric Anesthesiology, Nemours Children’s Health, Jacksonville, FL; ‡ Division of Pediatric Gastroenterology, Nemours Children’s Health, Jacksonville, FL.

**Keywords:** pediatric, endoscopy, gastroesophageal reflux disease, airway foreign body, complication

## Abstract

The Bravo pH probe is a wireless capsule allowing remote quantification of gastroesophageal reflux. A 14-year-old male presented for Bravo probe placement. Following esophagogastroduodenoscopy, attachment of the Bravo probe was attempted. Immediately, the patient began coughing without oxygen desaturation. Repeat endoscopy did not reveal the probe within the esophagus or stomach. He was then intubated, and fluoroscopy demonstrated a foreign body within the bronchus intermedius. Rigid bronchoscopy was performed to retrieve the probe using optical forceps. This is the first case of pediatric inadvertent airway deployment requiring retrieval. We recommend endoscopic visualization of the delivery catheter entering the cricopharyngeus before Bravo probe deployment, then followed by repeat endoscopy to confirm position of the probe after attachment.

Extended monitoring of esophageal pH remains the gold standard for diagnosis of gastroesophageal reflux (GER) in children (1). Traditional transnasal pH catheters are associated with discomfort and dysphagia. Furthermore, alterations in diet and activity during catheter tests may invalidate the results. The Bravo pH system (Medtronic, Shoreview, MN) is a wireless capsule consisting of an antimony pH electrode, radiotransmitter, and battery that communicate data to an externally worn device (2). In children, the Bravo probe is routinely positioned in the esophagus proximal to the LES in conjunction with esophagoscopy. Following pH data collection, the Bravo probe detaches spontaneously from the mucosa after several days coursing the intestinal tract and ultimately excreted via stool.

## CASE HISTORY

A 14-year-old male presented with a history of GER. Two years prior, the patient was noted to have dental enamel erosions and was placed on a trial of omeprazole. He had occasional heartburn and intermittent regurgitation, but no dysphagia or pharyngeal burning sensation.

The patient underwent flexible esophagogastroduodenoscopy (EGD). General anesthesia was induced via propofol infusion and spontaneous ventilation maintained with nasal cannula.

Esophageal findings included longitudinal furrows, edema, and mucosal friability. Cold forceps biopsies were performed, and a measurement was made from the incisors to 6 cm proximal to the lower esophageal sphincter (LES). Following standard Bravo deployment consisting of pH probe attachment via suction and pinning, the patient immediately began coughing without oxygen desaturation. Repeat endoscopy did not reveal the Bravo probe in the esophagus or stomach.

**FIGURE 1.**
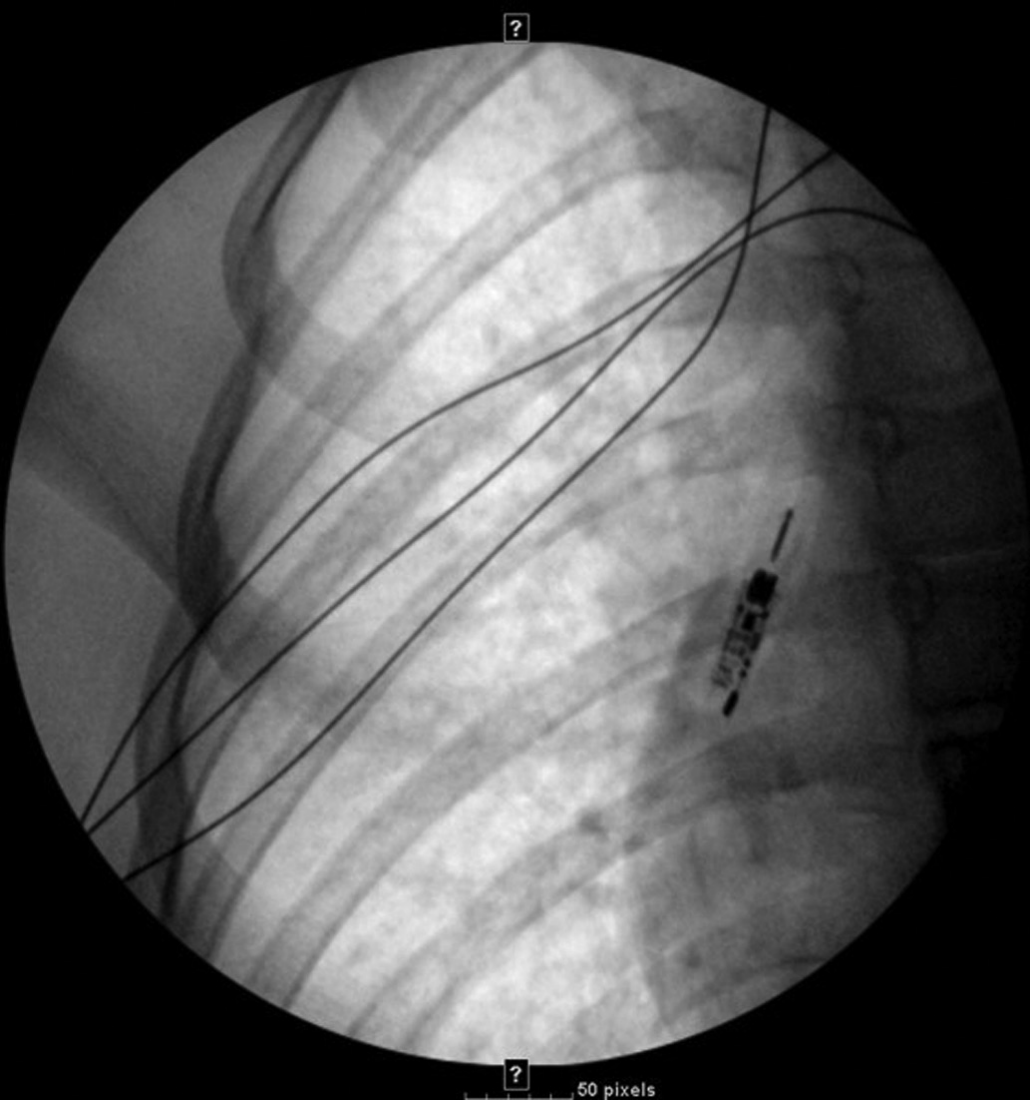
Intraoperative single-view fluoroscopy of the right chest demonstrated a metallic foreign body within the right bronchus intermedius.

Anesthesia was deepened using propofol and rocuronium, and the patient was intubated orally. A fluoroscopic chest film demonstrated a metallic foreign body overlying the right main stem bronchus (Fig. 1), and a member of the pediatric otolaryngology service was consulted.

**FIGURE 2.**
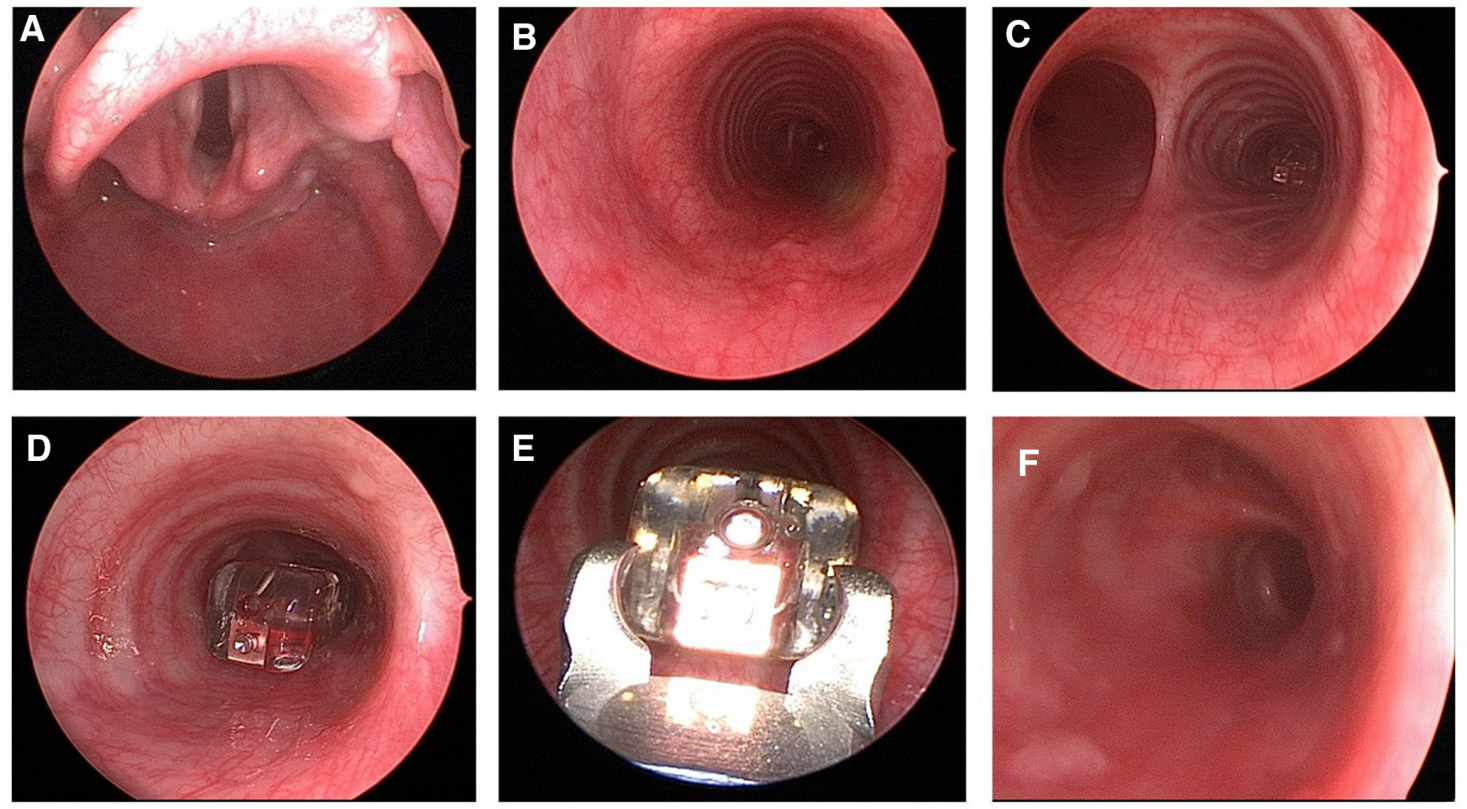
Intraoperative microlaryngoscopy and bronchoscopy findings. A) Supraglottis. B) Subglottis and trachea. C) Carina. D) Bronchus intermedius with Bravo probe attached to posterior wall. E) Optical forceps used for retrieval. F) Bronchus intermedius after retrieval.

Microlaryngoscopy and bronchoscopy were then performed revealing the Bravo probe within the bronchus intermedius (Fig. 2A-D). A rigid bronchoscope and peanut-style optical forceps were used for extraction (Fig. 2E). The bronchus intermedius was again visualized without evidence of trauma, and the patient emerged from anesthesia without difficulty (Fig. 2F). The patient recovered well without tachypnea, coughing, or desaturation. Esophageal biopsies demonstrated esophagitis consistent with GER, and proton pump inhibitor therapy was restarted. After consultation with the family, the Bravo probe was not replaced, and no pH probe GER data were obtained.

## DISCUSSION

The design of the Bravo hardware includes a 6 × 5.5 × 25 mm^3^ capsule mounted to a catheter delivery system (2). During endoscopy, a measurement 6 cm from the LES to the incisors is made, the esophagoscope is then removed, and the delivery catheter is then introduced to the premeasured location. Suction is initiated to yield adequate esophageal entrapment when pressure exceeds 510 mmHg for 10–15 seconds, followed by activation of a spring-loaded pin to complete attachment. Repeat endoscopy ensures adequate placement.

Overall, the Bravo probe is tolerated well. In an adult study of 85 participants, 96% of subjects recorded esophageal acid exposure for 24 hours, and 89% recorded over 36 hours (3).

Three patients required endoscopic removal: 2 for pain and a single patient for failure to selfexpel. In a similar study, the Bravo probe demonstrated decreased pharyngeal discomfort compared with catheter-based systems (4%–14% vs. 73%–95,% respectively) (2). However, the Bravo probe is associated with more esophageal discomfort (33%–34% vs. 17%–18%, respectively) (2).

The Bravo probe is well tolerated in children. A study of 66 pediatric participants demonstrated successful deployment in all subjects (1). The Bravo was associated with decreased throat pain compared with a transnasal catheter (44% vs. 94%, respectively) (1). However, like the above adult studies, chest pain was more common with the Bravo probe (39% vs. 6%) (1). Children with the Bravo probe were more likely to resume normal activity, have normal appetite, and report increased satisfaction. Conversely, the Bravo probe is more costly than transnasal catheter systems. Transnasal systems may also be placed without anesthesia when concomitant upper endoscopy is not indicated.

Major complications related to the Bravo probe are rare. FDA reported complications include gastrointestinal bleeding, esophageal perforations not requiring surgical repair, dislodgement of the probe into the pharynx, and aspiration of the probe (4). Presumed aspiration of a probe was reported in a 44-year-old patient who began to retch and cough with oxygen desaturation to 74% immediately after Bravo deployment (5). Repeat endoscopy did not demonstrate the probe within the esophagus or stomach, and it was ultimately identified in the nasopharynx on lateral skull radiograph. The authors assumed the Bravo probe was aspirated and then coughed up into the nasopharynx.

Another adult patient was reported to experience a Bravo probe in the hypopharynx (6). Following deployment, repeat endoscopy failed to localize the Bravo probe within the esophagus or stomach. During withdrawal of the endoscope, the probe was identified within the left pyriform sinus of the hypopharynx. Because of the potential for Bravo probe displacement to the airway the patient was urgently evaluated by an interdisciplinary team (otolaryngology, pulmonary and anesthesia) to insure safe removal with foreign body removal forceps after endotracheal intubation. An otolaryngologist successfully retrieved the probe without complication.

These case reports involving adult patients share similarities with our pediatric patient. The first adult patient experienced respiratory distress with retching, cough, and desaturation. It is unclear if the authors suspected the probe was successfully deployed in the esophagus, dislodged, coughed up and then aspirated versus deployed directly into the airway. The second adult patient did not experience significant respiratory distress making it unlikely the Bravo probe was attached directly to the airway. It is also unclear as to whether the Bravo was dislodged using the endoscope, and there was no obvious reason for movement of the probe to the left pyriform sinus. In our case, the temporal relationship between placement and coughing made direct deployment to the airway most likely.

Ours is the first pediatric case of Bravo pH probe deployment in a bronchus, attached to the posterior wall. It was successfully and atraumatically retrieved from the patient’s airway using optical forceps. After careful review, we have implemented minor alterations in technique within our hospital. In the past, we performed EGD on children with general anesthesia with spontaneous ventilation via nasal cannula and a propofol infusion. Now in patients scheduled for Bravo probe placement, the plan before patient sedation is for an interdisciplinary team (anesthesia and gastroenterology) to discuss the potential need for endotracheal intubation or laryngeal mask airway (LMA). Our practice preference leans toward endotracheal intubation when airway instrumentation is required to avoid repeated deflations of the LMA cuff to allow the esophagoscope and catheter to pass, which may temporarily inhibit ventilation.

Following endoscopy and measurement from the LES, the endoscope is removed. The Bravo delivery probe is then placed to the predetermined distance. However, before suction attachment, the endoscope is reintroduced into the hypopharynx to ensure the delivery system enters the cricopharyngeus. This ensures esophageal probe placement before deployment. We then confirm position of the probe after attachment with repeat endoscopy.

## ACKNOWLEDGMENTS

Written informed consent was obtained from the family on behalf of the pediatric patient to share case details, imaging and bronchoscopic findings.
